# Influence of serum uric acid on bone and fracture risk in postmenopausal women

**DOI:** 10.1007/s40520-024-02819-2

**Published:** 2024-08-01

**Authors:** María-Jesús Gómez-de-Tejada-Romero, Carmen Murias-Henríquez, Pedro Saavedra-Santana, Nery Sablón-González, Delvys Rodríguez Abreu, Manuel Sosa-Henríquez

**Affiliations:** 1 Department of Medicine, University of Seville, Seville, Spain; 2 University of Las Palmas de Gran Canaria, Osteoporosis and Mineral Metabolism Research Group. Las Palmas de Gran Canaria, Canaria, Spain; 3 Canary Health Service, Insular University Hospital, Bone Metabolic Unit, Las Palmas de Gran Canaria, Canaria, Spain

**Keywords:** Uric acid, Bone mineral density, Calcaneus ultrasounds, Fragility fractures

## Abstract

**Aims:**

Uric acid has been associated with several metabolic conditions, including bone diseases. Our objective here was to consider the relationship between serum uric acid levels and various bone parameters (bone mineral density, ultrasonographic parameters, vitamin D, PTH and serum calcium), as well as the prevalence and risk of fragility fracture.

**Methods:**

An observational and cross-sectional study carried out on 679 postmenopausal women, classified into 3 groups according to their serum uric acid levels, in whom bone densitometry, calcaneus ultrasounds, PTH, vitamin D and serum calcium analysis were done. Bone fractures were collected through the clinical history and lateral spinal X-ray.

**Results:**

Higher uric acid levels were found in women with older age, high BMI, diabetes, and high blood pressure. Higher levels of PTH and serum calcium were also observed, but did not effect on vitamin D. Serum uric acid was positively related to densitometric and ultrasonic parameters and negatively associated with vertebral fractures.

**Conclusions:**

In the population of postmenopausal women studied, sUA levels were correlated with BMD, BUA, and QUI-Stiffness, and this correlation was independent of age and BMI. In addition, sUA was associated with a decrease in vertebral fractures. These results imply a beneficial influence of sUA on bone metabolism, with both a quantitative and qualitative positive effect, reflected in the lower prevalence of vertebral fractures.

## Introduction

Uric acid, a product of the degradation of purines, is part of the complex network of human metabolism and has been related to obesity, diabetes, and arterial hypertension [1–3]. Along with these metabolic diseases, a role of serum uric acid (sUA) in bone metabolism has also been pointed out in recent years, since its antioxidant action (in physiological ranges) could prevent bone loss and, consequently, osteoporosis (OP) [4].

However, ot all studies confirm this. It is true that many of them have shown that there is a positive correlation between serum levels within the physiological range of sUA and bone mineral density (BMD), the main factor for the diagnosis of OP [5–9]. For some, this relationship is a consequence of a common element, fatty tissue [10], while others give relevance to this correlation directly, finding some causality independent of fatty tissue [7]. However, a recent study found no link between BMD and sUA [11]. Furthermore, various studies have been carried out to ascertain the effect that the different levels in the physiological range of sUA could have on the production of fragility fractures, obtaining varied results [12–15].

On the other hand, quantitative ultrasound (QUS) is a measurement technique of bone structure, so that its parameters (BUA, SOS and Stiffness) are considered indirect indicators of bone quality [16, 17], one of the determining aspects of bone status. We have only found two studies that observed the effect of uric acid levels on these ultrasonographic parameters [18, 19] and with different results.

In this study, carried out in a large number of postmenopausal women (to eliminate bias by sex and hormonal status), our objectives were: first, to observe the relationship between sUA levels and BMD measured by DXA and the QUS parameters (as indicators of bone quantity and quality, respectively), as well as to see if there is an influence of sUA levels on relevant hormonal parameters in bone metabolism; considering in all this the effect of fatty tissue (as the main confounding variable and represented by the body mass index -BMI-). Secondly, we observed the effect of serum uric acid levels on the prevalence and risk of fragility fracture.

## Materials and methods

This is an observational, cross-sectional study carried out on 679 postmenopausal women treated at the Bone Metabolic Unit of the Hospital University Insular, Gran Canaria, Spain, from January 1st, 2018 to December 31st, 2019. For all subjects a questionnaire, previously validated and used in other similar clinical studies, was completed to gather clinical data. Body weight (in kg) and body height (cm) were measured to the nearest 0.1 kg and 0.1 cm, respectively, using a SECA-marked stadiometer. Weight was measured with light clothes. The BMI as weight/height^2^ (kg/m^2^) was calculated for each individual. Obesity was defined as a BMI ≥ 30. The diagnosis of type 2 diabetes mellitus (T2DM) was made following the criteria of The American Diabetes Association [20]. The patients had been referred to the bone metabolic unit for assessment and at the time of the first visit had not received any treatment that could affect either serum uric acid levels or bone mineral metabolism.

### Serum biochemical measurements: sample collection and laboratory techniques

Serum biochemical parameters were measured from blood samples collected in the early morning, after a fasting night, in the appropriate specific tubes for each determination, with the least possible venous compression and centrifuged at 1,500 g for 10 min. The serum was separated into aliquots and stored within one hour of extraction at -20 °C until the biochemical analyzes were carried out, although most were done on the same day as the extraction.

Serum uric acid, creatinine and calcium were analyzed by a biochemical automatic analyzer Cobas^®^ 8000 (Roche Diagnostics, Switzerland), in which normal levels are: uric acid: 3.5–7.2 mg/dL; creatinine: 0.67–1.17 mg/dL); and calcium: 8.5–10.5 mg/dL. Serum calcium was corrected by total proteins based on the formula:

### Corrected calcium (mg/dL): serum calcium (mg/dL) / [Total proteins (g/dL) /16 + 0.55

Serum levels of 25(OH) vitamin D were measured by immunochemical luminescence, according to the Nichols method (Nichols Institute Diagnostics, San Clemente, California, USA). Serum parathyroid hormone (PTH) concentrations for the intact molecule were determined by immunochemical luminiscence, according to the Nichols Advantage method (PTH normal values: 15–88 pg/mL).

Creatinine clearance (CCr) was calculated using the Cockcroft-Gault formula:

$$\:CCr=\frac{\left(140-Age\right)\times\:Weight\left(Kg\right)\times\:0.85}{72\times\:\left[SerumCreatinine\left(mg/dL\right)\right]}$$

Estimated Glomerular Filtration Rate (GFR) was calculated using MDRD-4 IDMS formula:

$$\eqalign{{\rm eGFR }& =175\:\times \:({\rm serum\:creatinine }/\text{88,4}{)}^{-\text{1,154}} \cr & \times \:(age{)}^{-\text{0,203}}\:\times \:\text{0,742}}$$

### Bone mineral density (BMD)

BMD was measured by dual x-ray absorptiometry (DXA), both in lumbar spine (L2-L4) and proximal femur, with a Hologic Discovery^®^ densitometer, (Hologic Inc. Waltham, USA). All the measurements were made by the same operator, so there was no inter-observer variation. BMD values were done as g/cm^2^.

Diagnosis of osteoporosis was based on the WHO densitometric criteria (BMD T-score ≤ − 2.5 in at least one of the anatomic sites, including the lumbar spine, the femoral neck and the total hip).

### Fragility fractures

#### Vertebral fractures

A lateral thoracic-lumbar X-ray was carried out on the subjects. All the X-rays were collated and studied by two different observers: one was a radiologist and the second was an expert on bone metabolic diseases. According to the Genant criteria, the existence of vertebral deformity was recorded when there was a reduction in the vertebral height higher than 20% [21].

#### Non-vertebral fractures

The remaining fragility fractures were confirmed by hospital clinical reports, from the emergency services or by radiography study, excluding the patients’ self-diagnosis of fractures. Given the small number of hip fractures observed, we decided to include these in the group of “non-vertebral fractures” since their individualized analysis was not statistically powerful.

### Quantitative ultrasound (QUS) measurements

All subjects underwent calcaneus measurement by QUS. This was carried out using the Sahara Clinical sonometer (Hologic Inc., Bedford, MA) which measures 3 parameters at a fixed region of interest in the mid-calcaneus: broadband ultrasound attenuation (BUA); speed of sound (SOS); and quantitative ultrasound index (QUI), a combination of BUA and SOS resulting in the formula:

$$\:QUI\hspace{0.17em}=\hspace{0.17em}0.41\:X\:(BUA\hspace{0.17em}+\hspace{0.17em}SOS)\:-\hspace{0.17em}571$$

### Ethics

The study was carried out in accordance with the Declaration of Helsinki [22] and was approved by the Ethics Committee of the Insular University Hospital. All patients were informed of the study objectives and their informed consent was requested.

### Statistical analysis

*Univariate analysis.* Categorical variables are expressed as frequencies, percentages and continuous as mean and standard deviation (SD) when data followed a normal distribution, or as median and interquartile range (IQR = 25th − 75th percentile) when distribution departed from normal. The percentages were compared, as appropriate, using the Chi-square ($\:{\chi\:}^{2}$) test or the exact Fisher test, the means by the t-test and the medians by the Wilcoxon test for independent data.

*Additive models for the bone markers.* For each of the DXA and QUS markers, a multidimensional analysis was performed in which, in addition to uric acid, age, BMI, diabetes mellitus status and vitamin-D were entered as co-variates. First, a variable selection based on the best subset and then Akaike Information Criterion (AIC) were conducted. Once the variables were selected, the eventual nonlinear effect of the continuous variables was explored by the additive models using cubic splines. The final models were summarized, in addition to *P*-values, in coefficients and standard errors (SE) for linear effects or cubic splines together with 95% confidence bands (95% CI).

*Logistic models for vertebral and fragility fractures.* For each of the binary factors, vertebral fractures versus non-fractures and fragility fractures versus non-fractures, two multivariate logistic analyses were carried out. In the first, the variables age, BMI, sUA (continuous scale), T2DM, CCr and Vitamin D were entered. In the second analysis, BMD markers were added. In both analyses, a selection of variables based on the best subset regression and Akaike Information Criterion (AIC) was then performed [3]. The models were summarized as p-values (likelihood ratio test) and odds-ratio, which were estimated by means 95% CI.

Statistical significance was set at *p* < 0.05. Data were analyzed using the R package, version 3.6.1 (R Development Core Team, 2019).

## Results

To consider the behavior of the different variables studied in relation to sUA levels, the study participants were grouped into 3 groups according to levels. The cut-off points were set taking into account that most of the women had normal sUA levels. Thus, so that a similar number and homogeneous of patients in the 3 groups could be obtained, they were grouped into: low levels (below 4 mg/dl; medium levels (from 4 to 5 mg/dl); and high levels (above 5 mg/dl) (Tables 1 and 2).

**Table 1:** Characteristics of the women: overall and according to the level of serum uric acid

		Levels of serum uric acid			
	**Overall** *N* = 913	**< 4 mg/dL** *N* = 341	**4–5 mg/dL** *N* = 277	**> 5 mg/dL** *N* = 295	***P*** **-value**
Age (years)	61.4 ± 13.4	59.8 ± 13.2	59.9 ± 13.5	64.7 ± 13.0	< 0.001
BMI (kg/m^2^)	27.3 ± 5.4	25.6 ± 4.7	27.4 ± 5.2	29.2 ± 5.5	< 0.001
Obesity	270 (29.6)	64 (18.8)	86 (31.1)	120 (40.7)	< 0.001
Fractures					
By fragility (all)	298 (32.6)	108 (31.7)	81 (29.2)	109 (37.0)	0.129
Vertebral	103 (11.3)	52 (15.2)	21 (7.6)	30 (10.2)	0.009
Non vertebral	226 (24.8)	70 (20.5)	69 (24.9)	87 (29.5)	0.033
Uric acid (mg/dL)	4.5 ± 1.3	3.3 ± 0.5	4.4 ± 0.3	5.9 ± 1.0	< 0.001
Diabetes mellitus	125 (13.7)	35 (10.3)	36 (13.0)	54 (18.3)	0.012
Arterial hypertension	393 (43.0)	113 (33.1)	118 (42.6)	162 (54.9)	< 0.001
Urolithiasis	148 (16.3)	42 (12.4)	48 (17.4)	58 (19.7)	0.037
*Osteoporosis*	395 (44.0)	169 (50.1)	123 (44.9)	103 (35.9)	0.002
CCr (mL/min)	70.8 (56.5 ; 86.2)	71.9 (58.6 ; 87.1)	73.7 (62.4 ; 90.0)	65.3 (52.0 ; 82.3)	< 0.001
GFR (mL/min/1,73 m^2^)	73 (63 ; 84)	77 (69 ; 89)	74 (65 ; 85)	66 (53 ; 76)	< 0.001
25(OH) vitamin D (ng/mL)	22.4 (16.0 ; 30.0)	23.0 (16.0 ; 31.0)	21.9 (15.9 ; 29.7)	22.2 (16.0 ; 29.9)	0.501
PTH (pg/mL)	49.9 (36.0 ; 80.0)	43.0 (32.7 ; 59.4)	48.9 (36.8 ; 80.2)	62.8 (41.8 ; 100.8)	< 0.001
Corrected calcium (mg/dL)	9.9 (9.5 ; 10.3)	9.8 (9.4 ; 10.1)	9.9 (9.6 ; 10.3)	10.0 (9.6 ; 10.6)	< 0.001

Data are means SD, medias (IQR) and frequencies (%)

### Characteristics of the women according to the level of serum uric acid

The results showed that age and BMI were significantly higher in the groups with the highest levels of sUA, as well the presence of obesity, T2DM and arterial hypertension; nevertheless, osteoporosis diagnosis was lower. GFR and CCr were higher in the groups with lower sUA levels.

### Bone metabolism parameters

Regarding the variables related to bone metabolism, no significant differences were observed in 25(OH) vitamin D levels among the 3 groups, but PTH increased significantly in the groups with the highest levels of sUA (*p* < 0.001). This result was reflected in protein-corrected calcemia, which was higher in these same groups (*p* < 0.001).

### DXA and QUS parameters

The lumbar BMD and BUA levels were notably higher in the groups with raised figures in sUA (*p* = 0.002 and 0.004, respectively) (Table 2).

**Fig. 1:**
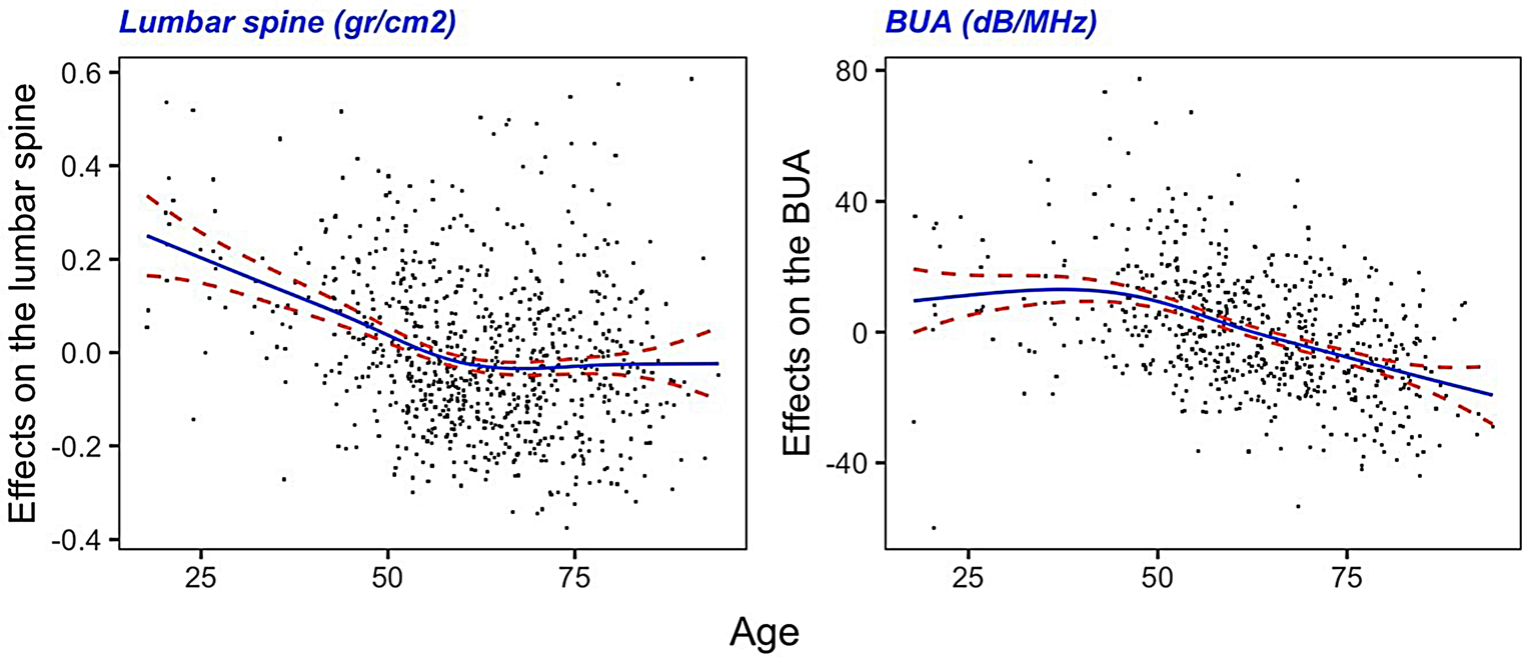
Cubic splines (95% CI) corresponding to the nonlinear effects of the age on the BMD in lumbar spine and the *BUA*. Note that the effects on the *femoral neck*, *Total Hip*, *SOS* and *Qui-Stiffness* were linear (see Table 2)

For each one of the DXA and QUS parameters, Table 3; Fig. 1 summarizes the additive regression models. For lumbar spine, total hip, BUA and Qui-Stiffness, uric acid showed significant linear association, adjusting for those co-variates that were selected by the best subset method and AIC (Age, BMI, sUA, DM2, CCr and Vitamin-D). In the models corresponding to lumbar spine and BUA, the effect of age on each of these markers was nonlinear. The corresponding cubic splines are shown in Fig. 1.

**Table:** Additive models for the *DXA* and *QUS* parameters

Bone marker	Covariates	Coefficients (SE)	*P*-value
*Lumbar spine* (g/cm^2^)	(Intercept)	0.5631 (0.0330)	< 0.001
	Age (years)	**Nonlinear effect**	< 0.001
	BMI (Kg/m^2^)	0.0079 (0.0011)	< 0.001
	**Uric Acid (mg/dL)**	**0.0159 (0.0044)**	**< 0.001**
	Vitamin-D ≥ 20	0.0230 (0.0113)	0.042
*Femoral neck* (g/cm^2^)	(Intercept)	0.6528 (0.0328)	< 0.001
	Age (years)	-0.0043 (0.0004)	< 0.001
	BMI (Kg/m^2^)	0.0077 (0.0010)	< 0.001
	**Uric Acid (mg/dL)**	**0.0057 (0.0032)**	**0.071**
	Vitamin-D ≥ 20	0.0237 (0.0078)	0.003
	CCr	0.0005 (0.0002)	0.022
*Total hip* (g/cm^2^)	(Intercept)	0.6531 (0.0318)	< 0.001
	Age (years)	-0.0045 (0.0003)	< 0.001
	BMI (kg/m^2^)	0.0135 (0.0009)	< 0.001
	**Uric Acid (mg/dL)**	**0.0072 (0.0036)**	**0.046**
	Vitamin-D	0.0010 (0.0004)	0.007
*BUA* (dB/MHz)	(Intercept)	35.0588 (3.8507)	< 0.001
	Age (years)	**Nonlinear effect**	< 0.001
	BMI (Kg/m^2^)	0.6401 (0.1363)	< 0.001
	**Uric Acid (mg/dL)**	**1.9139 (0.5356)**	**< 0.001**
*SOS (*m/s)	(Intercept)	1585.9 (8.88)	< 0.001
	Age (years)	-1.3444 (0.1161)	< 0.001
	BMI (Kg/m^2^)	1.3253 (0.2778)	< 0.001
	CCr	-0.2070 (0.0719)	0.004
*Qui-Stiffness*	(Intercept)	99.9 (4.68)	< 0.001
	Age (years)	-0.6840 (0.0546)	< 0.001
	BMI (Kg/m^2^)	0.5529 (0.1493)	< 0.001
	**Uric Acid (mg/dL)**	**1.3696 (0.5879)**	**0.02**

*Age*, *BMI*, *Uric Acid* (continuous scale), *Diabetes Mellitus*, *CCr* and *Vitamin-D* were entered in all analysis

Selection of variables were carried out using the best subset regression method and the AIC.

The effects of the selected covariates on the marker are shown for each of the bone markers. When the effects were nearly linear (effective degree of freedom ~ 1), the effect was considered linear. The nonlinear effects are shown in Fig. 1

**Table 3:** DXA and US markers adjusted by age and BMI *(*according to the level of serum uric acid)

	Levels of uric acid			
	< 4 mg/dL	4–5 mg/dL	> 5 mg/dL	*p*-value
***Spine lumbar***				0.002
g/cm^2^	0.839 [0.822 ; 0.857]	0.851 [0.832 ; 0.870]	0.886 [0.867 ; 0.905]	
T-score	-1.932 [-2.100 ; -1.764]	-1.818 [-2.002 ; -1.635]	-1.478 [-1.661 ; -1.296]	
***Femoral Neck***				0.588
g/cm^2^	0.675 [0.663 ; 0.687]	0.672 [0.659 ; 0.685]	0.682 [0.669 ; 0.695]	
T-score	-1.511 [-1.621 ; -1.402]	-1.537 [-1.656 ; -1.418]	-1.451 [-1.570 ; -1.332]	
***Total Hip***				0.153
g/cm^2^	0.789 [0.775 ; 0.803]	0.798 [0.782 ; 0.813]	0.810 [0.794 ; 0.825]	
T-score	-1.344 [-1.489 ; -1.199]	-1.252 [-1.410 ; -1.094]	-1.128 [-1.286 ; -0.970]	
***BUA***				0.004
dB/MHz	58.9 [56.6 ; 61.3]	60.2 [57.8 ; 62.5]	64.3 [62.0 ; 66.7]	
T-score	-1.187 [-1.334 ; -1.040]	-1.107 [-1.255 ; -0.959]	-0.846 [-0.992 ; -0.701]	
***SOS***				0.207
m/s	1520.7 [1516.8 ; 1524.6]	1522.9 [1518.9 ; 1526.8]	1525.8 [1521.9 ; 1529.7]	
T-score	-1.470 [-1.598 ; -1.342]	-1.398 [-1.527 ; -1.270]	-1.304 [-1.430 ; -1.177]	
***Qui-Stiffness***				0.097
Measure	77.0 [74.5 ; 79.6]	77.4 [74.9 ; 80.0]	80.7 [78.2 ; 83.2]	
T-score	-1.411 [-1.550 ; -1.273]	-1.390 [-1.529 ; -1.251]	-1.213 [-1.349 ; -1.076]	

Adjusted means (95% CI) by *age* and *body mass index* (BMI) obtained by least squares regression

**Table 4:** Multivariate logistic analysis for *fragility fractures* and *vertebral fractures*

		Analysis 1		Analysis 2	
Outcome	Covariates	*P*-value	Odd-Ratio (95% CI)	*P*-value	Odd-Ratio (95% CI)
Vertebral fractures *	*Age*, per year	< 0.001	1.06 (1.04 ; 1.09)	< 0.001	1.04 (1.02 ; 1.07)
	*BMI*, per kg/m^2^	-	-	0.009	1.08 (1.02 ; 1.14)
	***Uric Acid***, **per mg/dL**	**0.006**	**0.78 (0.65 ; 0.94)**	**0.03**	**0.81 (0.66 ; 0.98)**
	*Total hip*, per g/cm^2^	-	-	< 0.001	0.00 (0.00 ; 0.02)
*Fragility fractures*	*Age*, per year	< 0.001	1.05 (1.04 ; 1.06)	< 0.001	1.03 (1.02 ; 1.05)
	*BMI*, per kg/m^2^	-	-	< 0.001	1.07 (1.03 ; 1.11)
	*Diabetes mellitus*	0.055	1.50 (0.99 ; 2.28)	0.05	1.56 (1.00 ; 2.42)
	*Total hip*, per g/cm^2^	-	-	< 0.001	0.01 (0.00 ; 0.05)
Non vertebral fractures**	*Age*, per year	< 0.001	1.05 (1.03 ; 1.06)	0.007	1.02 (1.01 ; 1.04)
	*BMI*, per kg/m^2^	-	-	0.003	1.06 (1.02 ; 1.10)
	*Diabetes mellitus*	0.048	1.61 (1.01 ; 2.56)	0.063	1.60 (0.98 ; 2.60)
	*Vitamin-D > 20*	0.099	0.74 (0.52 ; 1.06)	-	-
	*Femoral neck*, per g/cm^2^	-	-	< 0.001	0.02 (0.00 ; 0.12)

(*) Model for Vertebral Fractures versus Non-Fractures

(**) Model for Only Non-vertebral fractures versus Non-Fractures

The covariates entered in the first analysis were the Age, BMI, Uric Acid (continuous scale), Diabetes Mellitus, CCr and Vitamin-D and in the second analysis, **BMD markers were added**. Selection of variables was carried out using the AIC. P-value corresponding to the likelihood ratio test

### Multivariate logistic analyses for fractures

Multivariate logistic analyses for vertebral fractures versus non-fractures and fragility fractures versus non-fractures are summarized in Table 4. For the vertebral fractures, type one logistic analyses (not including BMD markers) showed that the factors with independent association with the outcome (AIC) were age (per year, OR = 1.06; 95% CI = 1.04–1.09) and uric acid level (per md/dL, OR = 0.78; 95% CI = 0.65–0.94). When BMD markers are added in the analysis (type 2), BMI and BMD at the total hip are added in the model according to the AIC. Uric acid levels were maintained in the model (per mg/dL, OR = 0.81: 95% CI = 0.66 ; 0. 98). For the fragility fractures, the variables selected for the first model are age (per year, OR = 1.05; 95% CI = 1.04 ; 1.06) and T2DM (OR = 1.50; 95% CI = 0.99–2.28). When BMD markers are added in the analysis, BMI and BMD at total hip are added in the model according to the AIC. Uric acid level showed no statistical association with this outcome in any of the analyses (AIC).

## Discussion

Our initial results were as expected: sUA is related to the metabolic syndrome and, therefore, to obesity (and its determinant, BMI), T2DM, and arterial hypertension, as other studies have shown [23–26], which is explained by various metabolic mechanisms [1].

On the other hand, the decrease in GFR produces an increase in CCr and sUA, but both GFR and CCr generally decrease with age, so sUA levels should increase with age (and vice versa).

### sUA and bone metabolism parameters

The women under study had higher mean PTH levels in the groups with the highest sUA. In parallel, calcium levels also increased with sUA. All were within normal limits, except for some cases of high PTH levels, in which hyperparathyroidism was secondary to very low levels of vitamin D. It should be noted that the vitamin D values found in the women studied were generally very low, considered insufficient (< 30 ng/mL) and even close to deficiency (values < 20 ng/mL). Other authors have reported similar results [6, 8, 9]. The higher levels of uric acid found in our study are related to a decrease in renal function (lower CCr and lower GFR), which leads to a lower production of 1,25 (OH)_2_ vitamin D, which in turn stimulates the production of PTH, increasing calcemia. There were no significant differences in 25(OH) vitamin D levels between the groups analyzed. Furthermore, we did not find a correlation between this parameter and uric acid, but this metabolite of vitamin D is not related to renal function, so it does not tell us anything in this sense. However, we could consider PTH as an indirect marker of 1,25 (OH)_2_ vitamin D status (and, therefore, of renal function).

### sUA and DXA and QUS parameters

Focusing on the bone parameters, we have obtained a positive association between sUA and the BMD values measured in the different locations, but the association was greater with lumbar BMD. Many different authors have linked sUA and BMD in studies conducted in different adult populations [5–10, 12, 13]. However, a recent report, conducted in a large number of adult men (*n* = 6704) in the United States of America, found no such association [11]. In another study, Dalbeth et al. [27] point out that the correlation between these 2 variables is not causal but coincidental, with confounding variables such as BMI, adiposity or hormonal status. Pirro et al. [10] also conclude in their study that the relationship between sUA and BMD is mediated by adiposity. Navipour et al. [5], in their study also conducted in men, did find a relationship between sUA and BMD, even when adjusting for age and BMI. Ibrahim et al. [8], in a study conducted in a large population of 2,981 healthy Qatari adults, only found a relationship between both variables in non-obese, young, and smoking women. In our study, as in that of Yan et al. [7], the association between BMD and sUA was independent of BMI (as additive regression models show), and sex and hormonal status were the same (all were postmenopausal women), so there do not seem to be any confounding variables in our correlation. Given that the influence is greater in the lumbar spine, this could be due to the increased metabolic activity in the trabecular bone, the main component of the vertebrae.

Of the 2 studies that linked sUA levels with the ultrasonographic parameters BUA, SOS and Qui-Stiffness, one of them was carried out only in men [18], finding a positive association. The other, carried out in both men and women, found an association in men, but not in women [19]. In our study, sUA levels were positively related to both BUA and Qui-Stiffness in postmenopausal women. These results do not coincide with the previous ones by Scitara et al. [19], who found no relationship between Qui-Stiffness (they do not assess BUA or SOS) and the levels of sUA in the women studied. Nor did they find a relationship between sUA and lumbar BMD and total hip in them, a relationship that we did find in our study. The authors justify this finding with the fact that their female population was predominantly premenopausal, and that at these ages the levels of sUA are lower and with a lower degree of variability than in postmenopausal women, therefore their influence on BMD and Qui-Stiffness is less noticeable. In our study, we note that the sUA levels were not associated with the ultrasound parameter SOS. The BUA parameter correlates with the BMD better than the SOS which could explain this fact [28, 29].

### sUA and fractures

Few published studies consider the influence of sUA on fracture risk. In addition, they are carried out in different populations, the majority in men [14, 15, 30], or in populations of people > 50 years of both sexes [13, 31, 32]. We have only found two studies conducted in postmenopausal women [12, 33], similar to ours. On the other hand, not everyone assesses the same type of fracture. The results of all of them differ: some find no relationship between levels of sUA and risk of fracture [13, 31]; others only relate them to hip fracture [32], but most find an inverse relationship between sUA levels and vertebral fractures [12, 14, 15, 33], as in our study. We found our results regarding fractures of great interest and observed that sUA is a variable that influences vertebral fractures, but not non-vertebral ones. However, this influence is enhanced by the BMD, as can be seen by including this parameter in the second analysis. Somehow these results are consistent with the greater direct association of sUA with lumbar BMD. Studies carried out with larger populations of age and sex obtain similar results [14, 15]. In a meta-analysis conducted by Yin et al. [34] the authors found an association between higher sUA levels and low overall fracture risk. This disparity in results is probably due to the different population groups studied in the aforementioned works. Our study has the strength of being carried out in a very homogeneous population (postmenopausal women) and with a number that allows statistical robustness. The fact that vertebral fractures are the most frequent in postmenopausal women would also explain our results. In addition, the more active metabolism of trabecular bone may be an important factor for uric acid to have a greater influence on the risk of vertebral fracture, although studies in this regard would be required to confirm this hypothesis.

## Conclusions

In the population of postmenopausal women studied, sUA levels were correlated with BMD, BUA, and QUI-Stiffness, and this correlation was independent of age and BMI. Regarding fractures, sUA was associated with a decrease in vertebral fractures. These results lead us to consider that there is a beneficial influence of sUA on bone metabolism, with a positive effect both quantitative (BMD) and qualitative (QUS), which is reflected in the lower prevalence of vertebral fractures.

## Data Availability

No datasets were generated or analysed during the current study.
